# HGFDB: a collective database of helmeted guinea fowl genomics

**DOI:** 10.1093/database/baaa116

**Published:** 2021-01-08

**Authors:** Xuzhen Li, Zhi Li, Quankuan Shen, Yunbin Pan, Xiao Dong, Zetan Xu, Shengchang Duan, Yunfei Li, Yuan Du, Shanshan Chen, Zhaocheng Ma, Yang Dong

**Affiliations:** Faculty of Animal Science and Technology, Yunnan Agricultural University, Kunming, Yunnan 650201, China; Nowbio Biotechnology Company, No. 168 Yunjing Road, Kunming, Yunnan 650201, China; State Key Laboratory for Conservation and Utilization of Bio-Resources in Yunnan, Yunnan Agricultural University, Kunming, Yunnan 650201, China; Faculty of Life Science and Technology, Kunming University of Science and Technology, Kunming, Yunnan 650093, China; State Key Laboratory of Genetic Resources and Evolution, Kunming Institute of Zoology, Chinese Academy of Sciences, Kunming, Yunnan 650201, China; Sino-Africa Joint Research Center, Chinese Academy of Sciences, Nairobi 999070, Kenya; Kunming College of Life Science, University of Chinese Academy of Sciences, Kunming, Yunnan 650201, China; Nowbio Biotechnology Company, No. 168 Yunjing Road, Kunming, Yunnan 650201, China; Nowbio Biotechnology Company, No. 168 Yunjing Road, Kunming, Yunnan 650201, China; Nowbio Biotechnology Company, No. 168 Yunjing Road, Kunming, Yunnan 650201, China; Nowbio Biotechnology Company, No. 168 Yunjing Road, Kunming, Yunnan 650201, China; Nowbio Biotechnology Company, No. 168 Yunjing Road, Kunming, Yunnan 650201, China; Nowbio Biotechnology Company, No. 168 Yunjing Road, Kunming, Yunnan 650201, China; College of Biological Big Data, Yunnan Agriculture University, Kunming, Yunnan 650201, China; Shanghai Yangjing-Juyuan Experimental School, No 333 Pucheng Road, Pudong, Shanghai 200120, China; State Key Laboratory for Conservation and Utilization of Bio-Resources in Yunnan, Yunnan Agricultural University, Kunming, Yunnan 650201, China; College of Biological Big Data, Yunnan Agriculture University, Kunming, Yunnan 650201, China; Key Laboratory for Agro-biodiversity and Pest Control of Ministry of Education, Yunnan Agricultural University, Kunming, Yunnan 650201, China

## Abstract

As a vigorous and hardy and an almost disease-free game bird, the domestic helmeted guinea fowl (*Numida meleagris,* hereafter HGF) has attracted considerable attention in a large number of genetic study projects. However, none of the current/recent avian databases are related to this agriculturally and commercially important poultry species. To address this data gap, we developed Helmeted Guinea Fowl Database (HGFDB), which manages and shares HGF genomic and genetic data. By processing the data of genome assembly, sequencing reads and genetic variations, we organized them into eight modules, which correspond to ‘Home’, ‘Genome’, ‘Re-sequence’, ‘Gene’, ‘Variation’, ‘Download’, ‘Tools’ and ‘Help’, HGFDB provides the most comprehensive view of the HGF genome to date and will be relevant for future studies on HGF structural and functional genomics and genetic improvement.

**Database URL:** http://hgfdb.ynau.edu.cn/

## Introduction

The domestic helmeted guinea fowl (*Numida meleagris*, NCBI Taxonomy ID: 8996, hereafter HGF), under the family of Galliformes, is an agriculturally important poultry species. Its domestication is generally recognized to originate from wild HGF in the southern part of the Sahara, particularly in West Africa (1, 2). As a kind of disease-resistant bird with strong adaptability, HGF can thrive in heterogeneous environment and is now reared commercially across continents in Europe, America and Asia. Due to its superior nutritional value and economic potential, the production of HGF is elevated rapidly to about 1.4% of the entire world’s poultry population, of which chicken takes 92.3%, duck takes 4.4% and turkey takes 1.8% (Food and Agriculture Organization Corporate Statistical, accessed 2018) (3). Besides being a valued source of meat, egg and feather, domestic HGF also serves as a physiological animal model in studying disease (4) and neuromuscular, mechanical and energetic strategies for locomotion (5, 6).

Although HGF is important, little genetic study has been experimented on it, especially when compared to chicken. Currently, much effort has been taken on exploration of the genetic variation of poultry species, which serves as an important first step to reveal the uniqueness and to identify valuable genetic resources. However, only a handful of papers have been published on genetic diversity of HGF, mainly by microsatellite markers (7) or mtDNA (8, 9). It was not until 2019 that the first draft HGF genome assembly obtained by short sequencing reads was published (10). Based on the third-generation long reads, optical and chromatin interaction mapping, our group has improved the HGF reference genome to the near-chromosome level with contig N50 of 68.3 Mb (Peng, M. *et al*., In press). Additionally, we have discovered a tremendous number of single nucleotide polymorphism and InDel markers for 129 re-sequenced samples, which provides a foundation for developing future sustainable genetic improvement and conservation programs.

Database is a primary source of information that allows users to achieve different research goals within the same dynamic system. Previously, several high-quality avian omics databases have been established, such as BirdBase (http://birdbase.arizona.edu/birdbase/) (11), Chicken QTLdb (https://www.animalgenome.org/cgi-bin/QTLdb/GG/index) (12), B10K (https://b10k.genomics.cn/) (13) and Chicken2K (http://chicken.ynau.edu.cn) (Peng, M. *et al*., In press). However, none of them are related to guinea fowls. Therefore, It is necessary to fill the data gap for researchers and breeders to use the information more efficiently. Here, we established and developed the first Helmeted Guinea Fowl Database (HGFDB, http://hgfdb.ynau.edu.cn/). It aims to provide a comprehensive and user-friendly data interface for HGF genomic resource to all researchers. The HGFDB provides a high-quality *de novo* genome assembly that approaches to near-chromosome level and 15 173 protein-coding genes (94.73% of which were functional annotated). Moreover, HGFDB offers variants information from 129 genomes embedded in ‘Variation’ search module. We also provide complete statistical report and analysis report for ‘Genome’ and ‘Re-sequence’ sections. The site’s navigation is based on data types. The website now contains prominent links to statics and analysis of genome and re-sequence of HGF. HGFDB provides widely used online tools such as search function, Blast and JBrowse. It is attractive for HGF researchers that HGFDB houses information of publications about HGF-relevant studies. Details about these publications were linked to PubMed (http://www.ncbi.nlm.nih.gov/pubmed) database. Additionally, details of publications from other journals but not presented in PubMed database were manually imported. All data are freely available for download in HGFDB Download page. The HGFDB aims to meet the needs of HGF research community, especially for studies on molecular biology, immune function, growth and overall production performance of HGF.

## Materials and methods

### Data content

Based on PacBio long and Illumina short read sequencing together with optical and chromatin interaction mapping, our group has *de novo* assembled a high-quality near-chromosome level genome assembly of the domesticated HGF. Whole-genome re-sequencing on 129 birds (Table S1) and RNA-seq of 10 tissues (i.e. heart, muscle, liver, spleen, lung, kidney, optic lobe, striatum, cerebral cortex and a mixture of brain tissues) were also carried out (Peng, M. *et al*., In Press). Using this assembly as the reference, genome annotation, variant calling and population genetic analysis were conducted. See the following sections for methods.

### Genome annotation

The HGFv1 assembly was annotated for gene content using the NCBI Eukaryotic Genome Annotation Pipeline (14). Tandem Repeats Finder (15) was used to search for tandem repeats in repeat annotation. We employed EVidence Modeler(16) to merge RNA sequencing data and protein alignments with gene predictions and homologous method annotation into the final gene set and performed protein alignments using Exonerate (17) and tblastn (18) with avian proteomes of *Anas platyrhynchos, Gallus gallus, Meleagris gallopavo* and *Taeniopygia guttata* for protein-coding gene prediction. Protein-coding gene function was assigned according to the best match using Blastp against Swiss-Prot, Translation of EMBL (19) and Kyoto Encyclopedia of Genes and Genomes (20). The InterProScan functional analysis and Gene Ontology IDs were obtained with InterProScan (21). The Gene Ontology enrichment was done with Ontologizer 2.0 (22) with a *P*-value cut-off of 0.05.

### Variants calling

For SNPs, the Genome Analysis Toolkit option was set as ‘QD < 4.0, QUAL < 30.0, FS > 60.0, MQ < 40.0, MQRankSum < −10.0, ReadPosRankSum < −7.0, ReadPosRankSum > 7.0, BaseQRankSum < −6.0, BaseQRankSum > 6.0, SOR > 3.0’. Cluster Size and ClusterWindowSize were set to 4 and 10, respectively. For the total variants including SNPs and indels, VCFtools (23) with argument ‘‐ ‐mac 1 ‐ ‐minDP 1 ‐ ‐max-missing 1’ was applied to obtain the final no-missing data set, which included 44 035 924 biallelic SNPs and 4 214 076 indels for 129 individuals.

### Phylogeny, genetic diversity, singleton, linkage disequilibrium decay and ROHs

The phylogenetic tree was constructed based on autosomal SNPs using the maximum-likelihood method implemented in FastTree v2 (24). The individual ancestry coefficients were calculated by ADMIXTURE v1.3 (25), with K value varying between 2 and 10. According to the Principal Components Analysis and ADMIXTURE results, populations were re-grouped. Furthermore, the comprehensive R package PopGenome (26) was applied to perform population genomic analyses, and the R package SeqVarTools (27) was used to count singletons per individual with countSingletons function. The runs of homozygosity (ROHs) were detected by R package detectRUNS (28) by using the pruned data set to eliminate the impact of strong linkage disequilibrium.

### Publication data collection

By using the query in NCBI PubMed: ((((((((((QTL[Title/Abstract]) OR gene[Title/Abstract]) OR genome[Title/Abstract]) OR map[Title/Abstract]) OR microsatellite[Title/Abstract]) OR annotation[Title/Abstract]) OR EST[Title/Abstract]) OR marker[Title/Abstract]) OR sequence[Title/Abstract]) OR GWAS[Title/Abstract]) AND ((guinea fowl [Title/Abstract]) OR Numida meleagris [Title/Abstract]), we imported a total of 548 publications to HGFDB.

### Implementation

**Figure 1.**
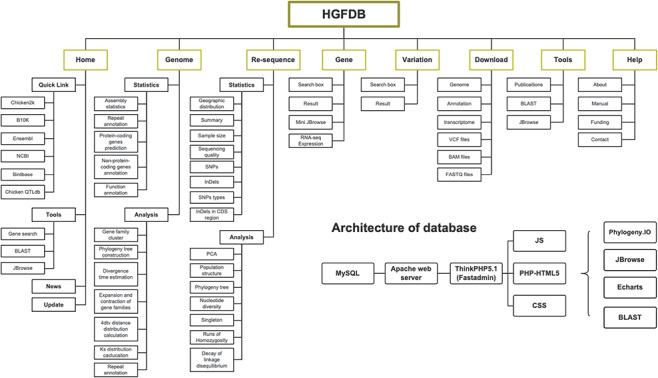
Framework of HGFDB. Eight main modules are highlighted in the dark yellow boxes. The corresponding contents of each module are listed below. The bottom right shows the architecture of HGFDB.

The HGFDB bases on Apache web server (http://www.apache.org), adopts ThinkPHP5.1 (http://www.thinkphp.cn)-based Fastadmin template, includes frameworks of Codelgniter (https://www.codeigniter.com/) and Bootstrap (https://getbootstrap.com), and applies programming language including CSS, PHP-HTML5 and JS. The MySQL (https://www.mysql.com) is used for data sorting, storage and management, and the AJAX asynchronous loading scheme is used for quick data loading and function implementation. To provide an interactive user experience, some interesting elements such Echarts (https://echarts.apache.org/zh/index.html), JBrowse (http://jbrowse.org), phylogeny.IO (https://github.com/oist/phylogeny-io) and BLAST server have been applied (Figure 1). The File Transfer Protocol–based () Download function is also offered with a transfer speed up to 50 Mbps. Additionally, all functions in HGFDB can be used normally on Safari, Chrome, Firefox, IE and Edge by mobile phone, pad and computer, respectively.

## Results

To organize various types of data and analyze the results, HGFDB is designated into eight main modules (Figure 1). The detailed description and function of each module can be found in the following sections.

### Home

‘Home’ module offers users an overview of HGFDB and some quick links to common databases, such as NCBI (29), Ensembl (30), Chicken2k, birdbase (11), B10K (13) and Chicken QTLdb (12), as well as links to more frequently used tools like quick search of gene information, BLAST and JBrowse. At the bottom of the page, we provide update logs and information of the related projects (Figure 1).

### Genome

**Figure 2.**
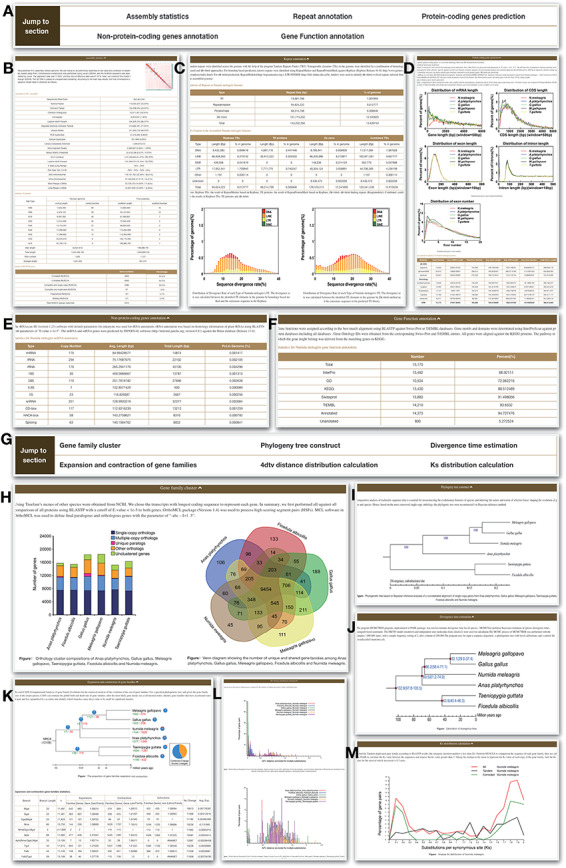
Genome module. Genome module has subpages, which are Statistics (A–F) and Analysis pages (G–M). (A) Navigation bar of the Statistics page, which provides a quick access to each section. (B) Assembly statistics. (C) Repeat annotation. (D) Protein-coding genes prediction. (E) Non-protein-coding genes annotation. (F) Functional annotation. (G) Navigation bar of the Analysis page. (H) Gene family cluster. (I) Phylogeny tree. (J) Divergence time. (K) Expansion and contraction of gene families. (L) 4dtv distance distribution. (M) Ks distribution.

‘Genome’ module has two sections, which are ‘Statistics’ and ‘Analysis’ (Figure 2). Five parts are included in the ‘Statistics’ section, which are assembly statistics, repeat annotation, protein-coding genes, non-protein-coding genes and functional annotation. The information will help user understand the HGFv1 genome more clearly. ‘Analysis section’ has some basic results, such as gene family cluster, phylogeny tree, divergence time, expansion and contraction of gene families, 4dtv distance distribution and Ks distribution.

### Re-sequence

**Figure 3.**
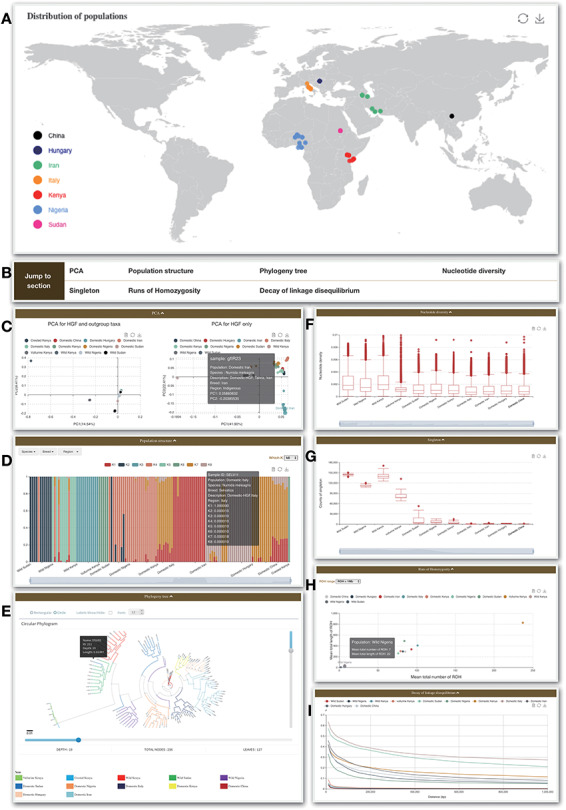
Re-sequence module. (A) Geological distribution of 129 individuals. In addition, there are some bar charts on this page. (B) Navigation bar of the Analysis page in the Re-sequence module. (C) The PCA section shows all 129 individuals belonging to HGF and outgroup taxa (left) and PCA for HGF only (right). (D) Bar plot of ADMIXTURE analysis for all samples with K from 2 to 10. (E) Phylogenetic tree. (F) Nucleotide diversity. (G) Singleton statistic. (H) ROHs. (I) Decay of linkage disequilibrium.

‘Re-sequence’ module contains two sections (Figure 3). ‘Statistics’ presents summaries for samples embedded in the database. The geographic distribution (Figure 3A), summary information, sample size and distribution, categories as well as sequencing depth and coverage are included. This information will help user to clearly see summary statistics for 129 samples. For convenience, ‘SNPs’, ‘Indels’, ‘SNPs types’ and ‘Indels in CDS region’ are added to this section.

In the ‘Analysis’ section, we set up the following seven parts and adopted dynamic and interactive charts, so that users can overview the information about 129 individuals more clearly.

1. The PCA part presents results for two datasets: all 129 individuals belonging to HGF and outgroup taxa, and the other is PCA for HGF only (Figure 3C).
2. The ADMIXTURE part indicates the proportions of proposed ancestry components in each sample by bar plots (Figure 3B). Length of each colored bar indicates the proportion of representative ancestry in each individual. The number of proposed ancestries is defined by ‘which K’. Choosing ‘Region’, ‘Category’ and/or ‘Purpose’ that can be determined by user shows different grouping results and includes ADMIXTURE clustering results for population with K from 2 to 10 (Figure 3D).
3. By presenting with Phylogeny.IO, the maximum likelihood tree of 129 samples constructed by FastTree (24) with 1000 bootstraps is present. The interactive interface allows user to format (rectangular or circle), drag and scale the tree. The information for node or leaf (i.e. sample) is shown in the pop-up window. Double-clicking on node will hide the certain branch (Figure 3E).
4. Nucleotide diversity part presents genetic diversity indexes across 11 guinea fowl populations (only one sample was excluded) and each population statistic is represented by boxplot (Figure 3F).
5. Singleton statistic is also displayed by boxplot (Figure 3G).
6. The ROHs part describes the distribution pattern of ROH in chicken and jungle fowl samples and populations. The results provide clues about the level of effective population size and demographic history (i.e. isolation, admixture, bottleneck and inbreeding) (54). The length of ROH can be characterized by ‘ROH range’ (Figure 3H).
7. Decay of linkage disequilibrium part presents the results of 11 populations (Figure 3I).

In addition, the small components equipped for each graph not only allows to filter dispaly data by clicking the legend, but also to present the data in text format by clicking the button in the upper right corner.

### Gene

**Figure 4.**
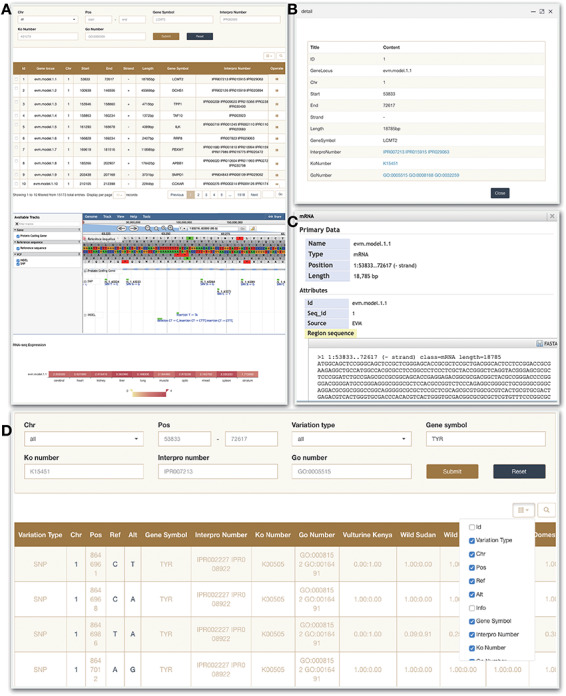
Gene and Variation modules. (A) Gene search module. (B) Click on operate button shows the detailed information. (C) Click on sequence in the JBrowse to view detailed information. (D) Variation module.

In this module, users can quickly search for the genes of interest, and get the corresponding information, such as structural annotations and functional annotations. Each entry contains gene locus, chromosome position, strand, length, gene symbol, InterPro number, KEGG number, Gene ontology number and operate pop-ups (Figure 4A and B). The number of the function annotation can be clicked to jump to the corresponding website. In addition, we have deployed a mini JBrowse window and RNA-seq expression part at the bottom of the page, which allows to view the location and variation of the target gene, the detailed sequence information by clicking (Figure 4C) as well as gene expression in 10 tissues.

### Variation

In the current version, the ‘Variation’ module contains 44 035 924 non-redundant SNPs and 4 214 076 Indels of 129 genomes in total. A search tool allows users to search according to several criteria. Thereinto, ‘Variation type’ box allows users to display only SNPs or InDels results, ‘Chr’ and ‘Pos’ locate the query interval. Moreover, users can search the target through the gene name. Other four search box provide different search type including ‘Gene symbol’ and three public databases number contain GO, KEGG and InterPro. In the search results, chromosome number, specific sites, specific mutant bases and related information of the target genes are provided (Figure 4D).

### Download

The ‘Download’ module makes HGFDB a valuable genomic resource for community. Currently, the reference genome, structure annotation and functional annotation are available, while the other types of data, such as raw genome sequencing data, RNA-seq data of 10 tissues, genetic variants information on scaffolds VCF format, sequence alignment BAM files and FASTQ files of 129 individuals, will be released soon.

### Tools

Currently, the ‘Tools’ module has integrated three tools, Publication, BLAST and JBrowse, which help users to focus on the target genomic region, find the literature and/or books related to guinea fowl and perform web-based BLAST with our assembly, respectively.

**Figure 5.**
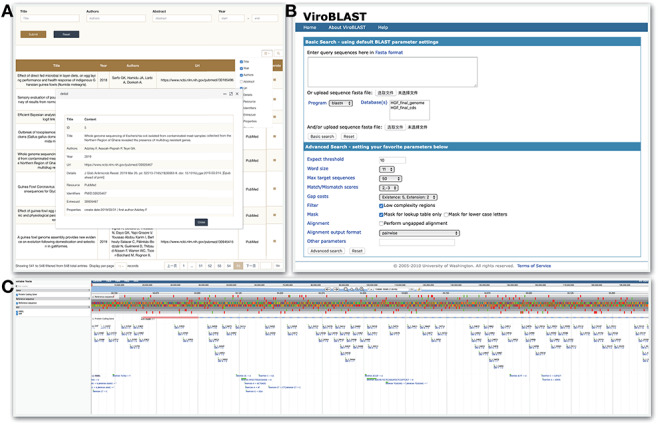
Tools module. HGFDB provides three tools, which are (A) Publication that allows to search related papers by keywords, (B) BLAST that compares users’ nucleotide or protein sequence with our assembly and annotation results and (C) JBrowse that provides the view of genomic features of HGF assembly.

1. The ‘Publication’ section contains a total of 548 literature or books. On searching keyword in title, abstract, author and/or year of publication, it shows a list and clicking on the target title pops up a new page with detailed information, such as abstract, publication type, etc. (Figure 5A).
2. The BLAST sequence similarity search server (Web-based BLAST server 2.2.28+) allows users to do the sequence alignment with our HGF genome assembly, coding sequence and protein sequences (Figure 5B) (31). The target sequence could be pasted or uploaded fasta format file. According to the selected program, proper sequences are listed in the database box for selection. After setting parameters and clicking on search bottom, the alignment result with overall alignment score, identities and percentage between query and subject sequence can be downloaded in HTML format.
3. As a combination of database and interactive web pages, JBrowse in HGFDB facilitates viewing sets of genomic and genetic features in different colors on our assembled genome HGFv1 (Figure 5C). In addition, by clicking on specific data entry, the detailed feature page of data entries can be reached. In the future updates, other interesting data, such as expression abundances derived from the RNA-seq data and messenger RNA, will be added to this genome browser.

### Help

In order to achieve a better user experience, we constructed the ‘Help’ module. It starts with a general introduction of HGFDB and includes a user manual that describes the content and function of each module. Additionally, the detailed contact information is provided.

## Conclusion and future plan

As the first database of HGF, HGFDB is a valuable resource for broad applications to HGF genetics and genomics study. The current implementation of HGFDB integrates data including germplasm information, genome assembly, genomic variation, genes and gene expression, providing a free access for data visualization, search and downloading.

With the advent of technology and decline of sequencing cost, the number of *de novo* assembly, re-sequencing and other omics studies for HGF is expected to keep increasing in the next few years. Therefore, a continuous effort will be made to ensure a most up-to-date follow-up of the HGF research progress. Future expansion plans of the database include improving the available information, cataloging functional genes, collecting detailed phenotypic data, etc. Development of cross-reference tools to compare data between HGF and other poultry species is also planned as a part of the future database expansion. Beyond this initial release, the overall aim of HGFDB is to provide long-term storage and support of the HGF research data and to provide informatic tools and services for access, data mining and knowledge discovery.

For the development of HGFDB, we welcome all kinds of comments and suggestions from users and researchers over the world. Through continuous updates, we believe that HGFDB can consequently facilitate and promote studies on HGF, which is an agriculturally and commercially important poultry species.

## Supplementary Material

baaa116_Supp

## References

[1] Larson G. and Fuller D.Q. (2014) The evolution of animal domestication. *Annu. Rev. Ecol. Evol. Syst.*, 45, 115–136. doi: 10.1146/annurev-ecolsys-110512-135813

[2] Marshall F. (2000) The origins and development of African livestock: archaeology, genetics, linguistics and ethnography.*Origins Spread Domest. Anim. East Afr.*, 191–221.

[3] Rischkowsky B. and Pilling D. (2007) The state of the World's animal genetic resources for food and agriculture– in Brief. Commission on Genetic Resources for Food and Agriculture, Food and Agriculture Organization of the United Nations,.

[4] Duffy D., Downer R. and Brinkley C. (1992) The effectiveness of Helmeted Guineafowl in the control of the deer tick, the vector of Lyme disease. *Wilson Bull.*, 104, 342–345.

[5] Higham T.E. and Biewener A.A. (2011) Functional and architectural complexity within and between muscles: regional variation and intermuscular force transmission. *Philos. Trans. R. Soc. B Biol. Sci.*, 366, 1477–1487. doi: 10.1098/rstb.2010.0359 PMC3130453 21502119

[6] Daley M.A. and Biewener A.A. (2006) Running over rough terrain reveals limb control for intrinsic stability. *Proc. Natl. Acad. Sci. U. S. A.*, 103, 15681. doi: 10.1073/pnas.0601473103 PMC1622881 17032779

[7] Kayang B.B., Inoue-Murayama M., Hoshi T. et al. (2002) Microsatellite loci in Japanese quail and cross-species amplification in chicken and guinea fowl. *Genet. Sel. Evol.*, 34, 233. doi: 10.1051/gse:2002006 PMC2705430 12081810

[8] Murunga P., Kennedy G.M., Imboma T. et al. (2018) Mitochondrial DNA D-Loop diversity of the helmeted guinea fowls in Kenya and its implications on HSP70 gene functional polymorphism. *Biomed. Res. Int.*, 2018, 1–12. 10.1155/2018/7314038 PMC6258102 30539018

[9] Adeola A.C., Ommeh S.C., Murphy R.W. et al. (2015) Mitochondrial DNA variation of Nigerian domestic helmeted guinea fowl. *Anim. Genet.*, 46, 576–579. doi: 10.1111/age.12324 26153528

[10] Vignal A., Boitard S., Thébault N. et al. (2019) A guinea fowl genome assembly provides new evidence on evolution following domestication and selection in galliformes. *Mol. Ecol. Resour.*, 19, 997–1014. doi: 10.1111/1755-0998.13017 30945415 PMC6579635

[11] Schmidt C.J., Romanov M., Ryder O. et al. (2008) Gallus GBrowse: a unified genomic database for the chicken. *Nucleic Acids Res.*, 36, 719–723. 10.1093/nar/gkm783 PMC2238981 17933775

[12] Hu Z.L., Fritz E.R. and Reecy J.M. (2007) AnimalQTLdb: a livestock QTL database tool set for positional QTL information mining and beyond. *Nucleic Acids Res.* doi: 10.1093/nar/gkl946 PMC1781224 17135205

[13] Koepfli K.-P., Paten B. and O’Brien S.J. (2015) The Genome 10K Project: a way forward. *Annu. Rev. Anim. Biosci.*, 3, 57–111. 25689317 10.1146/annurev-animal-090414-014900 PMC5837290

[14] Pruitt K.D., Brown G.R., Hiatt S.M. et al. (2014) RefSeq: an update on mammalian reference sequences. *Nucleic Acids Res.*, 42, 756. doi: 10.1093/nar/gkt1114 PMC3965018 24259432

[15] Benson G. (1999) Tandem repeats finder: a program to analyze DNA sequences. *Nucleic Acids Res.*, 27, 573–580. doi: 10.1093/nar/27.2.573 9862982 PMC148217

[16] Haas B.J., Salzberg S.L., Zhu W. et al. (2008) Automated eukaryotic gene structure annotation using EVidenceModeler and the Program to Assemble Spliced Alignments. *Genome Biol.*, 9, R7. doi: 10.1186/gb-2008-9-1-r7 PMC2395244 18190707

[17] Slater G.S.C. and Birney E. (2005) Automated generation of heuristics for biological sequence comparison. *BMC Bioinform.*, 6, 31. doi: 10.1186/1471-2105-6-31 PMC553969 15713233

[18] Gerts E.M., Yu Y.K., Agarwala R. et al. (2006) Composition-based statistics and translated nucleotide searches: improving the TBLASTN module of BLAST. *BMC Biol.*, 4, 41. doi: 10.1186/1741-7007-4-41 PMC1779365 17156431

[19] Boeckmann B., Bairoch A., Apweiler R. et al. (2003) The SWISS-PROT protein knowledgebase and its supplement TrEMBL in 2003. *Nucleic Acids Res.*, 31, 365–370. doi: 10.1093/nar/gkg095 12520024 PMC165542

[20] Tanabe M. and Kanehisa M. (2012) Using the KEGG database resource. *Curr. Protoc. Bioinf.*, Chapter 1, Unit 1.12. doi: 10.1002/0471250953.bi0112s38 22700311

[21] Zdobnov E.M. and Apweiler R. (2001) InterProScan - an integration platform for the signature-recognition methods in InterPro. *Bioinformatics*, 17, 847–848. doi: 10.1093/bioinformatics/17.9.847 11590104

[22] Bauer S., Grossmann S., Vingron M. et al. (2008) Ontologizer 2.0 - a multifunctional tool for GO term enrichment analysis and data exploration. *Bioinformatics*, 24, 1650–1651. doi: 10.1093/bioinformatics/btn250 18511468

[23] Danecek P., Auton A., Abecasis G. et al. (2011) The variant call format and VCFtools. *Bioinformatics.*, 27, 2156–2158. doi: 10.1093/bioinformatics/btr330 21653522 PMC3137218

[24] Price M.N., Dehal P.S. and Arkin A.P. (2010) FastTree 2 - approximately maximum-likelihood trees for large alignments. *PLoS One*, 5, e9490. doi: 10.1371/journal.pone.0009490 PMC2835736 20224823

[25] Alexander D.H.D.H., Novembre J. and Lange K. (2009) Fast model-based estimation of ancestry in unrelated individuals. *Genome Res.*, 19, 1655–1664. 19648217 10.1101/gr.094052.109 PMC2752134

[26] Pfeifer B., Wittelsbürger U., Ramos-Onsins S.E. et al. (2014) PopGenome: an efficient Swiss army knife for population genomic analyses in R. *Mol. Biol. Evol.*, 31, 1929–1936. doi: 10.1093/molbev/msu136 24739305 PMC4069620

[27] Zheng X., Gogarten S.M., Lawrence M. et al. (2017) SeqArray-a storage-efficient high-performance data format for WGS variant calls. *Bioinformatics*, 33, 2251–2257. doi: 10.1093/bioinformatics/btx145 28334390 PMC5860110

[28] Biscarini F., Cozzi P., Gaspa G. et al. (2018) detectRUNS: detect runs of homozygosity and runs of heterozygosity in diploid genomes. *CRAN (The Comprehensive R Archive Network)*.

[29] Benson D.A., Cavanaugh M., Clark K. et al. (2017) GenBank. *Nucleic Acids Res.*, 45, D37–D42. 27899564 10.1093/nar/gkw1070 PMC5210553

[30] Zerbino D.R., Achuthan P., Akanni W. et al. (2018) Ensembl 2018. *Nucleic Acids Res.*, 46, D754–D761. 29155950 10.1093/nar/gkx1098 PMC5753206

[31] Bajgain P., Richardson B.A., Price J.C. et al. (2011) Transcriptome characterization and polymorphism detection between subspecies of big sagebrush (Artemisia tridentata). *BMC Genomics*, 12, 370. doi: 10.1186/1471-2164-12-370 PMC3150299 21767398

